# Dental caries and periodontitis risk factors in cleft lip and palate patients

**DOI:** 10.3389/fped.2022.1092809

**Published:** 2023-01-04

**Authors:** Qinrui Wu, Zhengyi Li, Yixin Zhang, Xian Peng, Xuedong Zhou

**Affiliations:** ^1^ State Key Laboratory of Oral Diseases & National Clinical Research Center for Oral Diseases, West China Hospital of Stomatology, Sichuan University, Chengdu, China; ^2^ Department of Cariology and Endodontics, West China Hospital of Stomatology, Sichuan University, Chengdu, China

**Keywords:** cleft lip and palate, oral hygiene, dental caries, periodontitis, alveolar bone deformity, tooth deformity, microbial dysbiosis

## Abstract

Cleft lip and palate (CLP) is the most common congenital facial malformation and has a significant developmental, physical, and psychological impact on those with the deformity and their families. Risk factors contributing to CLP may conclude as genetic factors and environmental factors. The anatomical and morphological abnormalities related to CLP are favorable for dental plaque accumulation on the tooth surface. Therefore, patients with CLP undergo poorer oral hygiene and higher susceptibility to dental caries and periodontitis. In this review, we aim to conclude and update probable causes underlying the association between CLP and poor oral health and provide novel ideas of targeted early prevention for such oral diseases.

## Introduction

**Figure 1:**
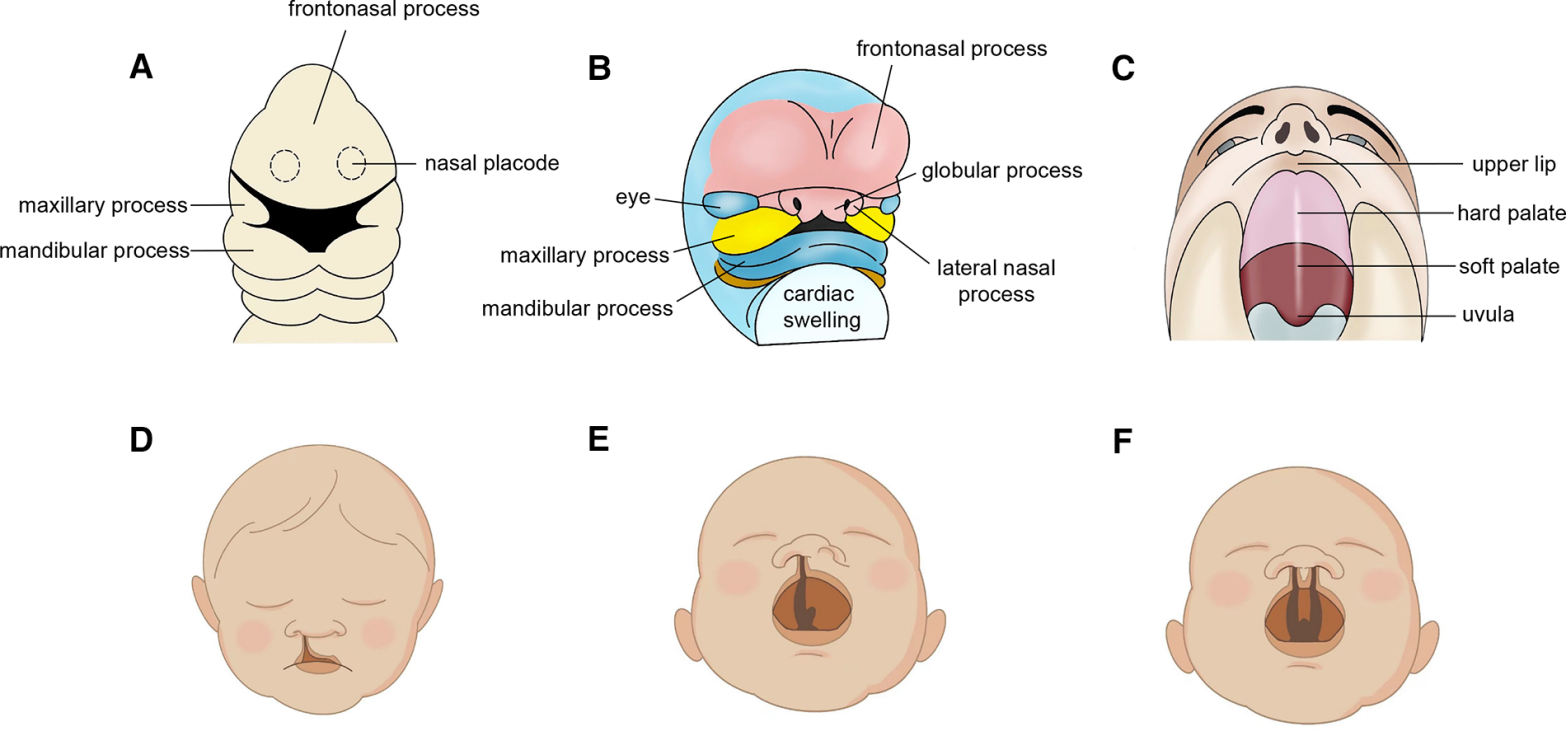
Embryonic development in oral and maxillofacial region. (**A**) embryonic week 4. (**B**) embryonic week 6. (**C**) developed lip and palate. Cleft phenotypes (**D**) Normal cleft lip. (**E**) unilateral cleft lip and palate (UCLP) and (**F**) bilateral cleft lip and palate (BCLP).

During embryogenesis, oral and maxillofacial areas are developed from frontonasal process, the paired lateral and medial nasal processes, and the paired maxillary processes. Cleft lip (CL) and cleft palate (CP) are two kinds of congenital malformation resulting from unsuccessful embryonic facial fusion processes and usually co-occur in the form of complete unilateral (UCLP) or bilateral (BCLP) cleft lip and palate (1). Based on their association with specific malformative patterns or their presence as isolated defects, CL/P can be classified as syndromic (SCLP) and non-syndromic, respectively (NSCLP) (2). Cleft lip and palate (CLP) affects roughly 10 million people worldwide and reduces quality of life, especially children and adolescents (3). Risk factors for facial cleft can be classified into four categories: genetic factors, pharmaceutical and physical factors, maternal metabolic or nutritional imbalances, maternal infection or injury (4) (Figure 1).

Children born with head and jaw deformations that affect the development and function of teeth and jaws are generally more susceptible to poor oral health (5). Dental caries and periodontitis are two most common infection-driven diseases in oral cavity prevalent in individuals with poor oral hygiene habits. Although the etiologies of caries and periodontitis are reported independent, with dental decay induced by supragingival plaque while periodontal infections by subgingival plaque (6), disruption of dynamic ecologic equilibrium in oral microbial biofilms can both lead to the onset of these two inflammatory diseases (7). Biofilms on tooth surface will produce acids in the presence of dietary carbohydrates. These acids demineralize the enamel and eventually allow cariogenic bacteria to invade the enamel, dentin and even the pulp (8). Subgingival biofilm destruct the structural integrity of the junctional epithelium, consequently inducing inflammation and thus leading to pocket formation (9).

Cleft lip and palate affect not only patients' appearance, but also disrupts the continuity of maxillary arch, affects tooth structure, shape, number, eruption and maxillofacial growth. Due to the adverse change of the anatomical and morphological alterations, patients with CLP and alveolar cleft are more likely to accumulate dental plaque in oral cavity. Thus, studies have reported that those children presented a higher prevalence of extensive dental caries and periodontitis compared with general population (10–12). The objective of the review is to summarize and update the body of evidence on the causes underlying the association between CLP and poor oral hygiene.

## Dental caries and periodontitis prevalence of children with CLP

Various epidemiological studies have demonstrated that there is an association between alveolar cleft and poorer oral health. A recent study found that among the children with cleft the prevalence of dental caries was found to be 71.9% (13). When comparing tooth caries and periodontal status, several studies showed that DMFT (Decayed, Missing, and Filled Teeth) and dmft scores were significantly higher in the control group without CLP, while plaque and gingiva indices such as plaque index and the gingival bleeding index were higher in CLP group than the controls (14–17). Different types of CLP seem to be quite diverged. Hazza'a et al. carried out a study and concluded that bilateral CLP experienced more dental caries than unilateral CLP patients, whereas only plaque accumulation was significantly higher in the BCLP patients (18). Howe et al. conducted a case-control study and recruited 3,326 patients with their relatives and confirmed the genetic factor is highly significant risk factor for children with CLP who are more susceptible to tooth decay (10). Evaluation of oral biofilm in children and adolescent revealed that the level of periodontal pathogens, including the most periodontopathic bacteria *Actinobacillus actinomycetemcomitans*, *Porphyromonas gingivalis*, and *Tannerella forsythia*, were statistically higher in the subgingival biofilms of the CLP group, thereby inducing more severe periodontitis (11, 19). Early-stage intervention such as early surgical repair is frequently performed within the first 1 to 2 years of life, which is of vital importance in team approach to cleft lip and palate (20). While fistulas following cleft palate repair may impair hygiene and occur in up to 35 percent of cases (21). Fritz et al. compared the oral health between patients with or without fistula after surgical correction of cleft palate and demonstrated a significantly lower oral health score in the fistula group, indicating poorer oral hygiene (22). Genes involved in the structure and development of congenital maxillofacial deformity may provide a basis for the link between CLP and dental caries. Mutations in EDARADD (ectodysplasin-A receptor-associated adapter protein) related with ectodermal dysplasia is likely to cause abnormal development of teeth, skin, hair, nails, and sweat glands (23). Therefore, the prevalence of dental caries was higher in syndromic cleft patients compared with those without a syndrome in northern Finland based on the outcomes of the current study (24).

**Figure 2:**
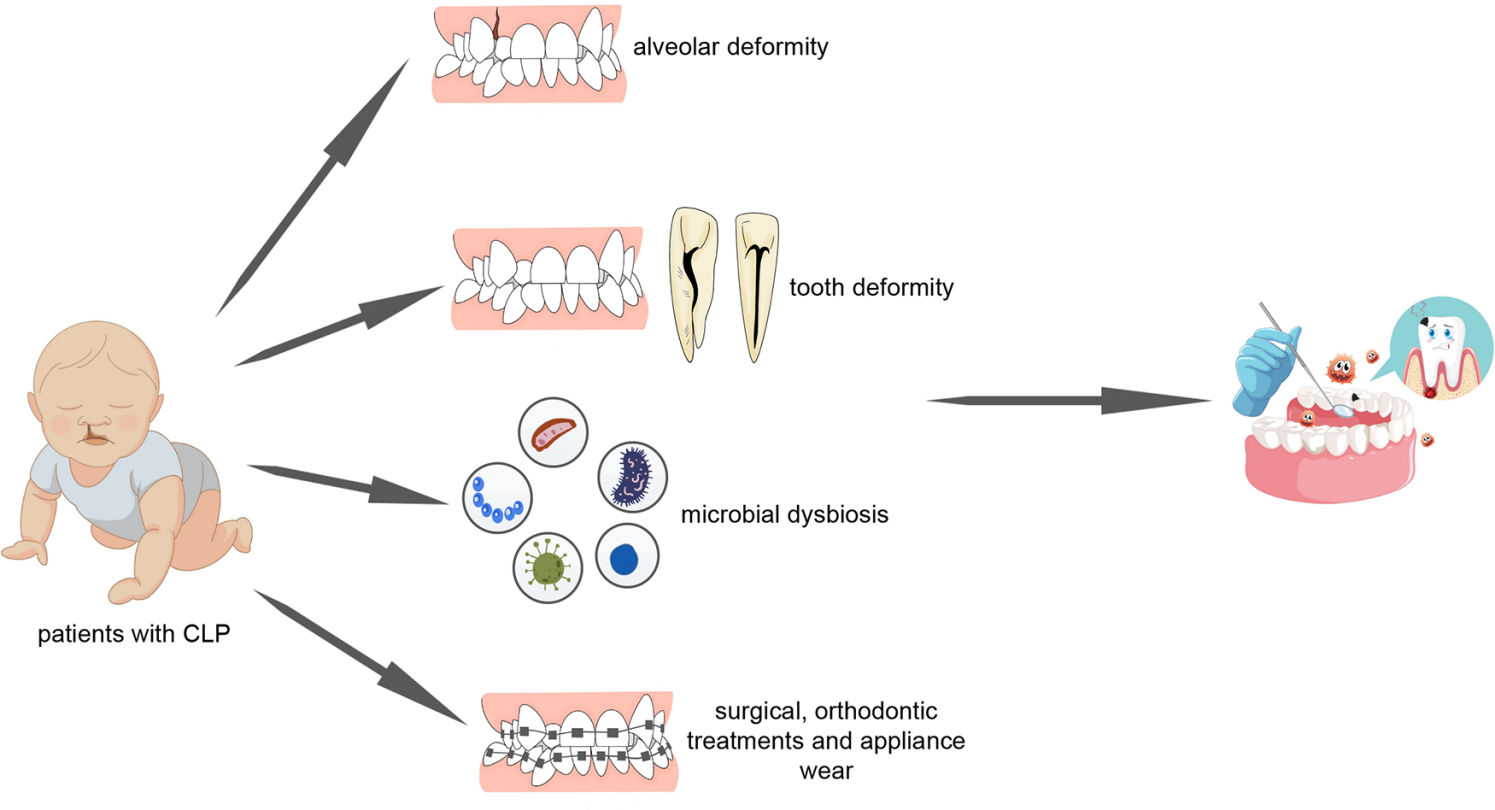
Patients with cleft lip and palate undergo poorer oral hygiene as a result of alveolar deformity, tooth deformity, microbial dysbiosis, surgical repair, orthodontic treatment and appliance wear.

Collectively, these studies allow us to confirm the hypothesis that children with cleft lip and palate have a significantly increased risk of dental caries and severe periodontal status as compared with the general population. Further in the text, we will explore the mechanisms that may contribute to such phenomenon (Figure 2).

## Mechanisms linking dental caries and CLP

### Tooth deformity

Children with oral clefts have a higher susceptibility for dental anomalies account for genetic and environmental factors. Numerous studies have demonstrated that dental anomalies are seen in a significant percentage of individuals with CLP (25–27). Cleft candidate genes such as MSX1, PAX9, IRF6, ANKS6 were confirmed associated with dental anomalies in individuals with CLP (28, 29). Methionine synthase gene responsible for methionine and homocysteine production may be associated with non-syndromic cleft lip and palate as well as dental caries (30). The structural deficiencies of embryonic development leads to microdontia, multiple missing teeth, ectopic teeth; supernumerary teeth; enamel and dentin malformation (27). Enamel hypoplasia often present with problems of discoloration and aesthetics, tooth sensitivity, reduced acid resistance, wear and erosion (31). Chapple et al. showed that developmental defects of enamel became more prevalent with age, with prevalence of 56% of 4-year-olds and 100% of 12-year-olds in patients with CLP (32). Kulas et al. carried out a case-control study and observed enamel color changes of permanent teeth three times more often in CLP children (33). While another research showed that there was no significant difference (*P* > 0.05) in the prevalence of development defects involving enamel among children with and without cleft (13). The rough surface attenuates acid resistant capacity of tooth and provides a critical niche for cariogenic bacteria to colonize. In addition, maxillary malformation, high incidence of supernumerary teeth and malposition of teeth result in crowding and dental malocclusion (34) which limits the use of toothbrush and antiseptic mouthwash and the natural cleaning of teeth by the tongue and saliva (35). Furthermore, tooth morphologic abnormalities like dens invaginatus also provide a favorable living environment for bacteria and fungi, which might be related to the pathogenesis and progression of dental caries (36, 37).

### Cariogenic microbial dysbiosis

Orofacial cleft affects the structures and functions of oral cavities is closely related to potentially harmful changes in the composition and activities of the oral microbiota (referred to as oral microbiota dysbiosis) (38). Some studies have demonstrated the fact that children with cleft may exhibit higher level of *Streptococcus mutans* (*S. mutans*), *Lactobacillus* and *Candida albicans* (*C. albicans*) in their saliva (39–41), which are the most common cariogenic microorganisms (42, 43). In a study determined *S. mutans* and *Lactobacilli* prevalence in children with CLP, *S. mutans* was detected in the saliva of 45% of the children and *Lactobacillus* in 16% with *S. mutans* in 48% of the plaque samples and *Lactobacilli* in 8% (44). A more recent study has collated and analyzed microorganisms in saliva of 80 subjects with CLP and 144 subjects without CLP using checkerboard DNA-DNA hybridisation technique. Although no statistically significance in salivary microbial profile was observed between two groups, low level of species diversity could provide us with microbiota dysbiosis in oral cavities of children with CLP (45). Current mechanistic study in Irf6 cKO animal models confirm that caries incidence/severity and bacterial load was increased in gene knockout mice. They found the number of total bacterial and *S. mutans* colony forming units was significantly higher in Irf6 cKO mice compared with mice of wildtype (46). Lastly, Rawashdeh et al. detected colonization rate of *Candida* was higher in patients with cleft than that in control group (47). However, the high level of *C. albicans* colonization in oral cavities of CLP patients is often related to denture or orthodontic appliance wear, the association between *C. albicans* colonization and CLP itself needs to be further exploration (48).

### Orthodontic treatments and appliance wear

Treatment of children and adolescents with CLP needs an organized team approach to provide optimal results. Orthodontic appliances have been linked to a higher caries experience (33). Fixed orthodontic appliances (FOAs) and dental prosthesis wear before or after surgical repair of CLP patients provides blind spots and reduces the effectiveness of saliva self-cleaning function (49). FOAs and denture base increases the area of plaque retention and makes it difficult for patients to maintain proper oral hygiene. The composition of dental plaque would change, leading to an increase in microbial population (50, 51), especially cariogenic bacteria *Streptococcus* and *Lactobacillus* which can lead to dental caries in the already susceptible mouths of individuals with CLP (52). van Loveren et al. carried out showed a comparative study and found that children performing early-stage preoperative orthopedics such as wearing acrylic plate in order to obturate the cleft were colonized earlier with *S. mutans* and *Lactobacillus* compared to non-plate oral cleft children. And the level of *S. mutans* increased with age (53)*.* A meta-analysis that combined 39 studies oral prostheses such as dentures/palatal obturators and FOAs may directly affect the growth of Candida in the oral cavity (48). Additionally, patients wearing such devices are more likely to eat soft food in order not to destroy orthodontic appliances, which may lead to food particles accumulation over tooth surface, and increase the susceptibility of dental caries.

## Mechanisms linking periodontitis and CLP

### Alveolar bone deformity

Alveolar bone defect, maxillary deformity and deformed dental arch can always be seen in CLP patients (54). CLP defects disrupt the continuity of maxillary dental arch. Study found that poor oral hygiene was found in all CLP patients, while the periodontal disease of patients with cleft lip, palate and alveolar cleft were more severe and might underwent more periodontal tissue destruction compared to patients with cleft palate only (55, 56). Huynh-Ba et al. carried out a 25-year comparative study and concluded that alveolar cleft sites were likely to have more periodontal tissue destruction, patients with orofacial cleft had higher levels of plaque and tended to lose more periodontal attachment at cleft site (57). A related study with 75 complete unilateral CLP patients design showed increased plaque index of teeth in the cleft region compared with teeth on the contralateral non-cleft side (58). Nevertheless, some other studies observed no statistically significance between different type of cleft (11, 47, 59), which need further exploration for lager sample sizes. Additionally, due to vestibule malformation in some CLP patients, the oppression of gingiva near cleft sites may influence oral hygiene, then adversely exacerbate mucogingival and periodontal status at discontinuous dental arches (47).

### Tooth deformity

CLP patients are born with a wide variety of dental anomalies, which could not only increase the susceptibility to dental caries, but also adversely affect periodontal status. Hypoplasia teeth were seen in 30.8% CLP subjects in a retrospective review (60). Crowded teeth favor the accumulation of dental plaque and make the removal difficult for CLP patients. In addition, dens invaginatus such as radicular lingual groove always found in maxillary lateral incisor has higher prevalence in patients with CLP (37), it creates a pathological dental pocket. Such a pocket resulted from an infolding of the enamel into dentine destroys the connection between periodontal tissue and cementum and allows bacteria propagate within, which can't be easily removed (26, 61, 62).

### Periodontal microbial dysbiosis

Periodontitis is a multifactorial inflammatory disease caused by microorganisms accumulating in dental plaques below the gingiva (63) and the initiation of periodontal diseases can be primarily summed up as commensal oral microbial dysbiosis (64). In the current literature, CLP patients have been reported different in microorganism profile (65). Early studies identified *Porphyromonas gingivalis*, *Actinobacillus actinomycetemcomitans* and *Tannerella forsythia* as causative agents in periodontal disease (66). Passinato et al. sampled dental plaque of individuals with or without CLP for subgingival microbiota analysis, they found higher level of those three periodontal pathogenic bacteria in the subgingival dental plaque of the CLP group, who underwent more severe gingivitis and periodontitis compared with control group (11). While Quirynene et al. did not find any significant differences of bacteria, neither in the proportion of aerobic and anaerobic bacteria among different sites (58). A more recent study described the subgingival microbial profile of children and adolescents with cleft and suggested higher relative proportion of Gram-negative anaerobic bacteria in teeth near the cleft compared with the control group (19). When comparing oral microbiota of infant with CLP and soft cleft palate (CSP), Machorowska et al. detected formation of the microbiota in individuals with CLP differed from that in CSP group, and the bacterial microenvironment was more pathogenic of patients with CLP, which is consistent with another comparative study of Tuna et al. (67). Such phenomenon could be explained by the communication between oral and nasal cavities and the transmission of bacteria from nasal cavity to oral cavity in CLP group (41).

When oral microbial dysbiosis takes place, pathogenic bacteria in oral cavity interacts with host immune system and induces inflammation and thus increases the risk of periodontal diseases. Taken as a whole, compared with individuals without CLP, periodonitits-related bacteria are more likely enriched in patients with CLP, which result in higher level of bacteremia and endotoxemia, consequently aggravating periodontitis.

### Surgical and orthodontic treatments

Children affected by CLP need multidisciplinary care from birth to adulthood and treatment of children and adolescents with CLP needs an organized team approach to provide optimal results. The early-stage surgical repair of CLP is central to current CLP team-approached treatment. However, some related technologies are still in their infancy and need further improvement and optimization (68). Some postoperative complications such as hypoplastic maxilla, concaved mid-face, deformed dental arch and oronasal fistula are common and difficult to completely circumvent after surgery (54, 69). Oronasal fistula occurs because of the poor wound healing after surgical repair and the overall incidence varied greatly from 0 to 77.8% (21). Fistula may cause nasal air escape, difficulty with pronunciation, food or liquid regurgitation, all of which may lead to communication between nasal and oral cavities and require repair (70). However, Karim et al. obtained a lower fistula rate using a modified palatoplasty technique (71). Such complications are likely to affect oral hygiene, promote dental plaque adherence to the teeth and influence periodontal health due to the patient's difficulty in tooth brushing around the fistula site. In addition, residual scar tissues after surgical repair constantly pull on their surrounding gingiva and could result in gingival recession along with speaking and swallowing, and thus make it easier for plaque accumulation (72). Notably, besides surgical procedures, combination of preoperative and postoperative orthodontic treatment play an indispensable role in team-approached treatment of CLP (73). Excessive orthodontic force may induce localized inflammatory responses and exacerbate the loss of periodontal attachment (55).

## Dental caries and periodontitis treatment effect of patients with CLP

Based on the ideas presented above, we can easily conclude that proper oral hygiene is difficult for many reasons, including anatomic factors, developmental factors, medical interventions, fear of pain, bleeding and soft tissue damage during toothbrushing near cleft site. Therefore, early dental interventions are necessary. Rivkin et al. found and stated that a regular brush may not be effective in the hygiene of the cleft area, the use of a small-sized toothbrush along with an interspace brush is efficient in some situations to improve oral hygiene of the patients with CLP mainly because the special toothbrush made it easier to reach interdental spaces (74).In other cases, selection of toothpastes, mouth rinses and use of fluoride products under the guidance of dentists could be helpful for improving oral hygiene (10, 75, 76). However, some studies found that there was no significant decrease of periodontal-related indices between CLP children with or without preventive dental care, suggesting nothing is more prior and effective than toothbrushing to remove dental plaque (77, 78). Novel biomaterials such as nanoparticulate cfDNA scavenger G3@SeHANs shows the therapeutic potential in periodontitis which is expected to be useful in CLP-related oral diseases and need to be further studied (79, 80).

## Summary

Given that the present state of knowledge suggests that children with CLP may have a greater risk factor for poor oral health than the general population. Risk factors include those related to the cleft itself and/or some complications of secondary surgical repair.

In healthy state, there is a well-balanced equilibrium between bacteria and the host. Maxillary bone or tooth malformation, bad dietary habits, incorrected toothbrushing technique of CLP patients may result in destroying the equilibrium (81), thereby increasing the susceptibility to periodontitis and dental caries. Dental caries and periodontitis are driven by a variety of pathogenic microorganism (7). However, much current studies have been limited to several bacteria or fungi detection. Further studies need to be carried out to include more kinds of microbial and compare microbiota profile between patients with or without CLP, even between different types of clefts.

In summary, poor oral hygiene-related dental caries and periodontitis are unfavorable for oral function, aesthetics and self-confidence of CLP patients. Although rehabilitation and some quality of life (QOL) instruments is possible with good quality care (82), orofacial clefts inevitably impose a burden to the individual, the family, and society. Therefore, families with CLP children are likely to participate in and strictly adhere to more health care programs and related services (83). Under such circumstances, parents of children with CLP should raise their awareness of oral health and receive preventive dental care as early as possible, which may reduce the risk of dental caries and periodontitis in the future.

## References

[1] Kožejová Jaklová L Hoffmannová E Dupej J Borský J Jurovčík M Černý M Palatal growth changes in newborns with unilateral and bilateral cleft lip and palate from birth until 12 months after early neonatal cheiloplasty using morphometric assessment. Clin Oral Investig. (2021) 25(6):3809–21. 10.1007/s00784-020-03711-9 33409695

[2] Mossey PA Little J Munger RG Dixon MJ Shaw WC. Cleft lip and palate. Lancet. (2009) 374(9703):1773–85. 10.1016/S0140-6736(09)60695-4 19747722

[3] Sandy J Davies A Humphries K Ireland T Wren Y. Cleft lip and palate: care configuration, national registration, and research strategies. J World Fed Orthod. (2020) 9(3s):S40–s4. 10.1016/j.ejwf.2020.09.003 33023731 PMC7532935

[4] Seifeldin SA. Is alveolar cleft reconstruction still controversial? (review of literature). Saudi Dent J. (2016) 28(1):3–11. 10.1016/j.sdentj.2015.01.006 26792963 PMC4688438

[5] Cheng LL Moor SL Ho CT. Predisposing factors to dental caries in children with cleft lip and palate: a review and strategies for early prevention. Cleft Palate Craniofac J. (2007) 44(1):67–72. 10.1597/05-112 17214528

[6] Loesche W. Dental caries and periodontitis: contrasting two infections that have medical implications. Infect Dis Clin North Am. (2007) 21(2):471–502. vii. 10.1016/j.idc.2007.03.006 17561079

[7] Sanz M Beighton D Curtis MA Cury JA Dige I Dommisch H Role of microbial biofilms in the maintenance of oral health and in the development of dental caries and periodontal diseases. Consensus report of group 1 of the joint EFP/ORCA workshop on the boundaries between caries and periodontal disease. J Clin Periodontol. (2017) 44(Suppl 18):S5–s11. 10.1111/jcpe.12682 28266109

[8] Hujoel PP Hujoel MLA Kotsakis GA. Personal oral hygiene and dental caries: a systematic review of randomised controlled trials. Gerodontology. (2018) 35(4):282–9. 10.1111/ger.12331 29766564

[9] Bosshardt DD. The periodontal pocket: pathogenesis, histopathology and consequences. Periodontol 2000. (2018) 76(1):43–50. 10.1111/prd.12153 29194796

[10] Howe BJ Cooper ME Wehby GL Resick JM Nidey NL Valencia-Ramirez LC Dental decay phenotype in nonsyndromic orofacial clefting. J Dent Res. (2017) 96(10):1106–14. 10.1177/0022034517709961 28535364 PMC5582684

[11] Passinato Gheller SA Porto AN Borba AM Veiga KA Aranha AMF. Periodontal findings in children and adolescents with cleft lip and/or palate: a case-control study. Pediatr Dent. (2021) 43(2):133–9. 33892839

[12] Worth V Perry R Ireland T Wills AK Sandy J Ness A. Are people with an orofacial cleft at a higher risk of dental caries? A systematic review and meta-analysis. Br Dent J. (2017) 223(1):37–47. 10.1038/sj.bdj.2017.581 28684841

[13] Chopra A Lakhanpal M Rao NC Gupta N Vashisth S. Oral health in 4-6 years children with cleft lip/palate: a case control study. N Am J Med Sci. (2014) 6(6):266–9. 10.4103/1947-2714.134371 25006561 PMC4083527

[14] Rocha MO Oliveira DD Costa FO Pires LR Diniz AR Soares RV. Plaque index and gingival index during rapid maxillary expansion of patients with unilateral cleft lip and palate. Dental Press J Orthod. (2017) 22(6):43–8. 10.1590/2177-6709.22.6.043-048.oar 29364378 PMC5784815

[15] Sundell AL Nilsson AK Ullbro C Twetman S Marcusson A. Caries prevalence and enamel defects in 5- and 10-year-old children with cleft lip and/or palate: a case-control study. Acta Odontol Scand. (2016) 74(2):90–5. 10.3109/00016357.2015.1044562 25972142

[16] Veiga KA Porto AN Matos FZ de Brito PC Borges ÁH Volpato LE Caries experience and periodontal status in children and adolescents with cleft lip and palate. Pediatr Dent. (2017) 39(2):139–44. 28390464

[17] Marzouk T Youssef M Tsigarida A McKinney C Wong C DeLucia L Association between oral clefts and periodontal clinical measures: a meta-analysis. Int J Paediatr Dent. (2022) 32(4):558–75. 10.1111/ipd.12934 34626516

[18] Hazza'a AM Rawashdeh MA Al-Nimri K Al Habashneh R. Dental and oral hygiene status in Jordanian children with cleft lip and palate: a comparison between unilateral and bilateral clefts. Int J Dent Hyg. (2011) 9(1):30–6. 10.1111/j.1601-5037.2009.00426.x 21226848

[19] Perdikogianni H Papaioannou W Nakou M Oulis C Papagiannoulis L. Periodontal and microbiological parameters in children and adolescents with cleft lip and /or palate. Int J Paediatr Dent. (2009) 19(6):455–67. 10.1111/j.1365-263X.2009.01020.x 19732188

[20] Preidl RHM Kesting M Rau A. Perioperative management in patients with cleft lip and palate. J Craniofac Surg. (2020) 31(1):95–101. 10.1097/SCS.0000000000005897 31633673

[21] Hardwicke JT Landini G Richard BM. Fistula incidence after primary cleft palate repair: a systematic review of the literature. Plast Reconstr Surg. (2014) 134(4):618e–27e. 10.1097/PRS.0000000000000548 25357056

[22] Fritz A Jodeh DS Qamar F Cray JJ Rottgers SA. Patients with a history of oronasal Fistula repair exhibit lower oral health measured with patient-centric outcomes measures. Cleft Palate Craniofac J. (2021) 58(9):1142–9. 10.1177/1055665620981331 33353404

[23] Shaffer JR Wang X Feingold E Lee M Begum F Weeks DE Genome-wide association scan for childhood caries implicates novel genes. J Dent Res. (2011) 90(12):1457–62. 10.1177/0022034511422910 21940522 PMC3215757

[24] Lehtonen V Sándor GK Ylikontiola LP Koskinen S Pesonen P Harila V Dental treatment need and dental general anesthetics among preschool-age children with cleft lip and palate in northern Finland. Eur J Oral Sci. (2015) 123(4):254–9. 10.1111/eos.12195 26031998

[25] Howe BJ Cooper ME Vieira AR Weinberg SM Resick JM Nidey NL Spectrum of dental phenotypes in nonsyndromic orofacial clefting. J Dent Res. (2015) 94(7):905–12. 10.1177/0022034515588281 26082386 PMC4530345

[26] das Neves LT de Carvalho IMM Cobourne MT Gomide MR. Dental anomalies in non-syndromic orofacial clefts: a clinical approach. Oral Dis. (2022) 28(5):1351–68. 10.1111/odi.14226 35485181

[27] Haque S Alam MK. Common dental anomalies in cleft lip and palate patients. Malays J Med Sci. (2015) 22(2):55–60. 26023296 PMC4438093

[28] Vieira AR Meira R Modesto A Murray JC. MSX1, PAX9, and TGFA contribute to tooth agenesis in humans. J Dent Res. (2004) 83(9):723–7. 10.1177/154405910408300913 15329380

[29] Letra A Fakhouri W Fonseca RF Menezes R Kempa I Prasad JL Interaction between IRF6 and TGFA genes contribute to the risk of nonsyndromic cleft lip/palate. PLoS One. (2012) 7(9):e45441. 10.1371/journal.pone.0045441 23029012 PMC3447924

[30] Mostowska A Hozyasz KK Jagodzinski PP. Maternal MTR genotype contributes to the risk of non-syndromic cleft lip and palate in the Polish population. Clin Genet. (2006) 69(6):512–7. 10.1111/j.1399-0004.2006.00618.x 16712703

[31] Seow WK. Developmental defects of enamel and dentine: challenges for basic science research and clinical management. Aust Dent J. (2014) 59(Suppl 1):143–54. 10.1111/adj.12104 24164394

[32] Chapple JR Nunn JH. The oral health of children with clefts of the lip, palate, or both. Cleft Palate Craniofac J. (2001) 38(5):525–8. 10.1597/1545-1569_2001_038_0525_tohocw_2.0.co_2 11522175

[33] Kulas A Illge C Bekes K Eckert AW Fuhrmann RA Hirsch C. Structural color changes in permanent enamel of patients with cleft lip and palate: a case-control study. J Orofac Orthop. (2016) 77(1):45–51. 10.1007/s00056-015-0007-z 26744208

[34] Suzuki A Nakano M Yoshizaki K Yasunaga A Haruyama N Takahashi I. A longitudinal study of the presence of dental anomalies in the primary and permanent dentitions of cleft lip and/or palate patients. Cleft Palate Craniofac J. (2017) 54(3):309–20. 10.1597/15-186 27031269

[35] Zou J Meng M Law CS Rao Y Zhou X. Common dental diseases in children and malocclusion. Int J Oral Sci. (2018) 10(1):7. 10.1038/s41368-018-0012-3 29540669 PMC5944594

[36] Fonseca-Souza G de Oliveira LB Wambier LM Scariot R Feltrin-Souza J. Tooth abnormalities associated with non-syndromic cleft lip and palate: systematic review and meta-analysis. Clin Oral Investig. (2022) 26(8):5089–103. 10.1007/s00784-022-04540-8 35729285

[37] Ickow IM Zinn S Stacy JM Jr. Martin B Losee JE D'Alesio A Dens invaginatus in patients with cleft lip and palate: a case series. Cleft Palate Craniofac J. (2021) 58(11):1452–8. 10.1177/1055665621998534 33663245

[38] Pitts NB Zero DT Marsh PD Ekstrand K Weintraub JA Ramos-Gomez F Dental caries. Nat Rev Dis Primers. (2017) 3:17030. 10.1038/nrdp.2017.30 28540937

[39] Shashni R Goyal A Gauba K Utreja AK Ray P Jena AK. Comparison of risk indicators of dental caries in children with and without cleft lip and palate deformities. Contemp Clin Dent. (2015) 6(1):58–62. 10.4103/0976-237X.149293 25684913 PMC4319347

[40] Sundell AL Ullbro C Marcusson A Twetman S. Comparing caries risk profiles between 5- and 10- year-old children with cleft lip and/or palate and non-cleft controls. BMC Oral Health. (2015) 15:85. 10.1186/s12903-015-0067-x 26208495 PMC4514989

[41] Machorowska-Pieniążek A Mertas A Skucha-Nowak M Tanasiewicz M Morawiec T. A comparative study of oral Microbiota in infants with complete cleft lip and palate or cleft soft palate. Biomed Res Int. (2017) 2017:1460243. 10.1155/2017/1460243 28393073 PMC5368409

[42] Struzycka I. The oral microbiome in dental caries. Pol J Microbiol. (2014) 63(2):127–35. 10.33073/pjm-2014-018 25115106

[43] Du Q Ren B He J Peng X Guo Q Zheng L Candida albicans promotes tooth decay by inducing oral microbial dysbiosis. Isme j. (2021) 15(3):894–908. 10.1038/s41396-020-00823-8 33149208 PMC8026629

[44] Bokhout B van Loveren C Hofman FX Buijs JF van Limbeek J Prahl-Andersen B. Prevalence of Streptococcus mutans and lactobacilli in 18-month-old children with cleft lip and/or palate. Cleft Palate Craniofac J. (1996) 33(5):424–8. 10.1597/1545-1569_1996_033_0424_posmal_2.3.co_2 8891374

[45] Sundell AL Ullbro C Dahlén G Marcusson A Twetman S. Salivary microbial profiles in 5-year old children with oral clefts: a comparative study. Eur Arch Paediatr Dent. (2018) 19(1):57–60. 10.1007/s40368-018-0326-z 29392531

[46] Tamasas B Cox TC. Massively increased caries susceptibility in an Irf6 cleft lip/palate model. J Dent Res. (2017) 96(3):315–22. 10.1177/0022034516679376 27927890

[47] Rawashdeh MA Ayesh JA Darwazeh AM. Oral candidal colonization in cleft patients as a function of age, gender, surgery, type of cleft, and oral health. J Oral Maxillofac Surg. (2011) 69(4):1207–13. 10.1016/j.joms.2010.02.044 20691530

[48] Khan I Ahmad T Manzoor N Rizvi MA Raza U Premchandani S. Evaluating the role of local host factors in the candidal colonization of oral cavity: a review update. Natl J Maxillofac Surg. (2020) 11(2):169–75. 10.4103/njms.NJMS_161_20 33897176 PMC8051668

[49] Ohyama T. Prosthodontic considerations for patients with cleft lip and palate. Int Dent J. (1986) 36(3):140–5. 3533786

[50] Arikan V Kizilci E Ozalp N Ozcelik B. Effects of fixed and removable space maintainers on plaque accumulation, periodontal health, candidal and Enterococcus Faecalis carriage. Med Princ Pract. (2015) 24(4):311–7. 10.1159/000430787 26044443 PMC5588238

[51] Addy M Shaw WC Hansford P Hopkins M. The effect of orthodontic appliances on the distribution of Candida and plaque in adolescents. Br J Orthod. (1982) 9(3):158–63. 10.1179/bjo.9.3.158 6954991

[52] Sukontapatipark W el-Agroudi MA Selliseth NJ Thunold K Selvig KA. Bacterial colonization associated with fixed orthodontic appliances. A scanning electron microscopy study. Eur J Orthod. (2001) 23(5):475–84. 10.1093/ejo/23.5.475 11668867

[53] van Loveren C Buijs JF Bokhout B Prahl-Andersen B Ten Cate JM. Incidence of mutans streptococci and lactobacilli in oral cleft children wearing acrylic plates from shortly after birth. Oral Microbiol Immunol. (1998) 13(5):286–91. 10.1111/j.1399-302X.1998.tb00709.x 9807120

[54] Shi B Losee JE. The impact of cleft lip and palate repair on maxillofacial growth. Int J Oral Sci. (2015) 7(1):14–7. 10.1038/ijos.2014.59 25394591 PMC4817536

[55] Schultes G Gaggl A Kärcher H. Comparison of periodontal disease in patients with clefts of palate and patients with unilateral clefts of lip, palate, and alveolus. Cleft Palate Craniofac J. (1999) 36(4):322–7. 10.1597/1545-1569_1999_036_0322_copdip_2.3.co_2 10426598

[56] Salvi GE Brägger U Lang NP. Periodontal attachment loss over 14 years in cleft lip, alveolus and palate (CLAP, CL, CP) subjects not enrolled in a supportive periodontal therapy program. J Clin Periodontol. (2003) 30(9):840–5. 10.1034/j.1600-051X.2003.00390.x 12956661

[57] Huynh-Ba G Brägger U Zwahlen M Lang NP Salvi GE. Periodontal disease progression in subjects with orofacial clefts over a 25-year follow-up period. J Clin Periodontol. (2009) 36(10):836–42. 10.1111/j.1600-051X.2009.01469.x 19703238

[58] Quirynen M Dewinter G Avontroodt P Heidbüchel K Verdonck A Carels C. A split-mouth study on periodontal and microbial parameters in children with complete unilateral cleft lip and palate. J Clin Periodontol. (2003) 30(1):49–56. 10.1034/j.1600-051X.2003.300108.x 12702111

[59] Al-Wahadni A Alhaija EA Al-Omari MA. Oral disease status of a sample of Jordanian people ages 10 to 28 with cleft lip and palate. Cleft Palate Craniofac J. (2005) 42(3):304–8. 10.1597/03-161.1 15865466

[60] Al Jamal GA Hazza'a AM Rawashdeh MA. Prevalence of dental anomalies in a population of cleft lip and palate patients. Cleft Palate Craniofac J. (2010) 47(4):413–20. 10.1597/08-275.1 20590463

[61] Chaturvedula BB Muthukrishnan A Bhuvaraghan A Sandler J Thiruvenkatachari B. Dens invaginatus: a review and orthodontic implications. Br Dent J. (2021) 230(6):345–50. 10.1038/s41415-021-2721-9 33772187

[62] Zhu J Wang X Fang Y Von den Hoff JW Meng L. An update on the diagnosis and treatment of dens invaginatus. Aust Dent J. (2017) 62(3):261–75. 10.1111/adj.12513 28306163

[63] Curtis MA Diaz PI Van Dyke TE. The role of the microbiota in periodontal disease. Periodontol 2000. (2020) 83(1):14–25. 10.1111/prd.12296 32385883

[64] Kinane DF Stathopoulou PG Papapanou PN. Periodontal diseases. Nat Rev Dis Primers. (2017) 3:17038. 10.1038/nrdp.2017.38 28805207

[65] Zhou F Su Z Li Q Wang R Liao Y Zhang M Characterization of bacterial differences induced by cleft-palate-related spatial heterogeneity. Pathogens. (2022) 11(7). 10.3390/pathogens11070771 PMC9323727 35890015

[66] Zambon JJ. Periodontal diseases: microbial factors. Ann Periodontol. (1996) 1(1):879–925. 10.1902/annals.1996.1.1.879 9118283

[67] Tuna EB Topçuoglu N Ilhan B Gençay K Kulekçi G. Staphylococcus aureus transmission through oronasal fistula in children with cleft lip and palate. Cleft Palate Craniofac J. (2008) 45(5):477–80. 10.1597/06-247.1 18788867

[68] Campbell A Costello BJ Ruiz RL. Cleft lip and palate surgery: an update of clinical outcomes for primary repair. Oral Maxillofac Surg Clin North Am. (2010) 22(1):43–58. 10.1016/j.coms.2009.11.003 20159477

[69] Sakran KA Wu M Alkebsi K Mashrah MA Al-Rokhami RK Wang Y The sommerlad-furlow modified palatoplasty technique: postoperative complications and implicating factors. Laryngoscope. (2022). 10.1002/lary.30385. [Epub ahead of print] 36120931

[70] Mahajan RK Kaur A Singh SM Kumar P. A retrospective analysis of incidence and management of palatal fistula. Indian J Plast Surg. (2018) 51(3):298–305. 10.4103/ijps.IJPS_84_18 30983730 PMC6440358

[71] Sakran KA Yin J Yang R Elayah SA Alkebsi K Zhang S Early cleft palate repair by a modified technique without relaxing incisions. Cleft Palate Craniofac J. (2022) 10556656221135288. 10.1177/10556656221135288. [Epub ahead of print] 36300250

[72] de Almeida AL Esper LA Pegoraro TA do Valle AL. Gingival recession in individuals with cleft lip and palate: prevalence and severity. Cleft Palate Craniofac J. (2012) 49(1):92–5. 10.1597/10-052 21534842

[73] Chen YH Liao YF Chang CS Lu TC Chen KT. Patient satisfaction and quality of life after orthodontic treatment for cleft lip and palate deformity. Clin Oral Investig. (2021) 25(9):5521–9. 10.1007/s00784-021-03861-4 33683466

[74] Rivkin CJ Keith O Crawford PJ Hathorn IS. Dental care for the patient with a cleft lip and palate. Part 2: the mixed dentition stage through to adolescence and young adulthood. Br Dent J. (2000) 188(3):131–4. 10.1038/sj.bdj.4800410 10717999

[75] Freitas AB de Barros LM Fiorini JE Boriollo MF Moreira AN Magalhães CS. Caries experience in a sample of adolescents and young adults with cleft lip and palate in Brazil. Cleft Palate Craniofac J. (2013) 50(2):187–91. 10.1597/11-143 22250859

[76] Parapanisiou V Gizani S Makou M Papagiannoulis L. Oral health status and behaviour of Greek patients with cleft lip and palate. Eur Arch Paediatr Dent. (2009) 10(2):85–9. 10.1007/BF03321606 19627672

[77] Pradhan L Shakya P Thapa S Nakarmi KK Maharjan A Sagtani RA Prevalence of dental anomalies in the patient with cleft lip and palate visiting a tertiary care hospital. JNMA J Nepal Med Assoc. (2020) 58(228):591–6. 10.31729/jnma.5149 32968294 PMC7580371

[78] Dogan MC Serin BA Uzel A Seydaoglu G. Dental anxiety in children with cleft lip and palate: a pilot study. Oral Health Prev Dent. (2013) 11(2):141–6. 10.3290/j.ohpd.a29364 23534040

[79] Huang H Pan W Wang Y Kim HS Shao D Huang B Nanoparticulate cell-free DNA scavenger for treating inflammatory bone loss in periodontitis. Nat Commun. (2022) 13(1):5925. 10.1038/s41467-022-33492-6 36207325 PMC9546917

[80] Zhu X Chu C-J Pan W Li Y Huang H Zhao L. The correlation between periodontal parameters and cell-free DNA in the gingival crevicular fluid, Saliva, and plasma in Chinese patients: a cross-sectional study. J Clin Med. (2022) 11(23):6902. 10.3390/jcm11236902 36498477 PMC9741438

[81] Gürsoy UK Gürsoy M Könönen E Sintim HO. Cyclic dinucleotides in oral Bacteria and in oral biofilms. Front Cell Infect Microbiol. (2017) 7:273. 10.3389/fcimb.2017.00273 28680857 PMC5478684

[82] Chen N Shi B Huang H. Velopharyngeal inadequacy-related quality of life assessment: the instrument development and application review. Front Surg. (2022) 9:796941. 10.3389/fsurg.2022.796941 35402476 PMC8988257

[83] Lewis CW Jacob LS Lehmann CU. The primary care pediatrician and the care of children with cleft lip and/or cleft palate. Pediatrics. (2017) 139:e20170628. 10.1542/peds.2017-0628 28557774

